# Quantitative Analysis of BTF3, HINT1, NDRG1 and ODC1 Protein Over-Expression in Human Prostate Cancer Tissue

**DOI:** 10.1371/journal.pone.0084295

**Published:** 2013-12-27

**Authors:** Andrew J. Symes, Marte Eilertsen, Michael Millar, Joseph Nariculam, Alex Freeman, Maria Notara, Mark R. Feneley, Hitenedra R. H. Patel, John R. W. Masters, Aamir Ahmed

**Affiliations:** 1 Prostate Cancer Research Centre, Division of Surgery, University College London, London, United Kingdom; 2 The Queen’s Medical Research Institute, University of Edinburgh, Edinburgh, United Kingdom; 3 Department of Histopathology, University College London Hospital, London, United Kingdom; 4 Division of Surgery, Oncology, Urology and Women's Health, University Hospital of Northern Norway, Tromso, Norway; H. Lee Moffitt Cancer Center & Research Institute, United States of America

## Abstract

Prostate carcinoma is the most common cancer in men with few, quantifiable, biomarkers. Prostate cancer biomarker discovery has been hampered due to subjective analysis of protein expression in tissue sections. An unbiased, quantitative immunohistochemical approach provided here, for the diagnosis and stratification of prostate cancer could overcome this problem. Antibodies against four proteins BTF3, HINT1, NDRG1 and ODC1 were used in a prostate tissue array (> 500 individual tissue cores from 82 patients, 41 case pairs matched with one patient in each pair had biochemical recurrence). Protein expression, quantified in an unbiased manner using an automated analysis protocol in ImageJ software, was increased in malignant vs non-malignant prostate (by 2-2.5 fold, p<0.0001). Operating characteristics indicate sensitivity in the range of 0.68 to 0.74; combination of markers in a logistic regression model demonstrates further improvement in diagnostic power. Triple-labeled immunofluorescence (BTF3, HINT1 and NDRG1) in tissue array showed a significant (p<0.02) change in co-localization coefficients for BTF3 and NDRG1 co-expression in biochemical relapse vs non-relapse cancer epithelium. BTF3, HINT1, NDRG1 and ODC1 could be developed as epithelial specific biomarkers for tissue based diagnosis and stratification of prostate cancer.

## Introduction

Prostate carcinoma is a disease of the epithelium, the most common cancer in men and the cause of considerable morbidity and mortality [1]. Each year, over 30,000 men are diagnosed of prostate cancer and over 10,000 die of the disease in the UK. In 2010, 217,730 new cases of prostate cancer were diagnosed in the US, with 32,050 American males dying of the disease [2]. 

Diagnosis and prognosis of prostate cancer is based on tissue morphology from biopsies (~1 million procedures in USA and ~70,000 in the UK / year). First time biopsies identify cancer in 38% of cases whereas equivocal diagnosis or false negatives constitute ~25-30% of cases [3]. Descriptors of aggressiveness (e.g. Gleason grade) determine cancer management and therapy but have significant drawbacks and high variance, particularly for low grade cancers. Immunohistochemistry (IHC) is used in resolving equivocal diagnosis, however, most IHC biomarkers have been identified using subjective (scoring) analysis introducing large variability [4]. For organ confined disease prostate biopsy remains a critical clinical tool, however, there is a need to resolve false negatives and refine diagnosis. By identifying cancers that have good prognosis over-treatment could be reduced but there are no quantitative protein markers to enhance the quality of diagnosis. A reproducible, quantitative approach could greatly facilitate this process. 

Discovery of IHC markers, largely, and their analysis, is conducted by semi quantitative approaches (e.g. scoring of tissue sections stained with a chromo- or fluorophore) with large inter-observer errors [4,5]. Scoring of the intensity of a chromophore (or fluorophore) involves visual inspection followed by a score within a predetermined range by the experimenter. An approach based upon visual observation of patterned objects like mammalian tissue, with distinct architecture, or visual assesment of intensity of a light signal as done for IHC scoring, entails with severe limitations of perception of illuminance and judgment, generally [6] and for assessment of prostate carcinoma, specifically [7]. A further disadvantage of using a subjective scoring approach is that the selection of target proteins to be investigated gravitates towards the extremes (either highly expressed in cancer (e.g. EZH) or absent in cancer (e.g. cytokeratins 5 and 6). This means that a large number of significant biological changes that inevitably occur within these extremes are not picked up and cannot be used for the understanding of the mechanisms of carcinogenesis or developed as biomarkers for diagnosis, or stratification or prognosis of cancer. Quantitative IHC [8] can identify novel biomarkers in an unbiased, reproducible manner, and in conjunction with fluorescent probes could be useful for the latter. It is likely that not only the expression but co-localization of two or more proteins may also change as a result of disease or treatment [9,10]. A pre-requisite for prognosis of the disease based upon molecular, rather than morphological, criteria also requires robust and reproducible detection of protein expression in IHC sections that could be followed by quantifying co-localization changes in immunofluorescence [10]. 

In a previous study we developed a semi-automated, quantitative method to measure the expression of Wnt5A in prostate tissue [8] in a prostate tissue array (> 500 tissue cores from 82 patients, 41 case pairs matched with one patient in each pair had biochemical recurrence). The aim of this study was to employ the quantitative IHC approach to assess if this method could be used to identify putative cancer markers for diagnostic purposes. We chose four genes BTF3, NDRG1, HINT1 and ODC1 that are up-regulated in prostate carcinoma (oncomine.org; selection criteria: p=0.0001, 2 fold change and in top 10% of genes over-expressed in the overall dataset) in at least four normal vs cancer prostate gene expression analysis datasets [11–14]. We used quantitative immunohistochemistry [8] to show that the expression of BTF3, HINT1, NDRG1 and ODC1 proteins is increased in prostate cancer tissue and could serve as putative targets for the investigation of carcinogenesis or biomarkers for disease. We then employed a quantitative immunofluorescence approach to stratify prostate cancer using co-localization coefficients of BTF3, HINT1 and NDRG1.

## Results

### Quantitative immunohistochemical analysis of proteins over-expressed in prostate cancer tissue

**Figure 1:**
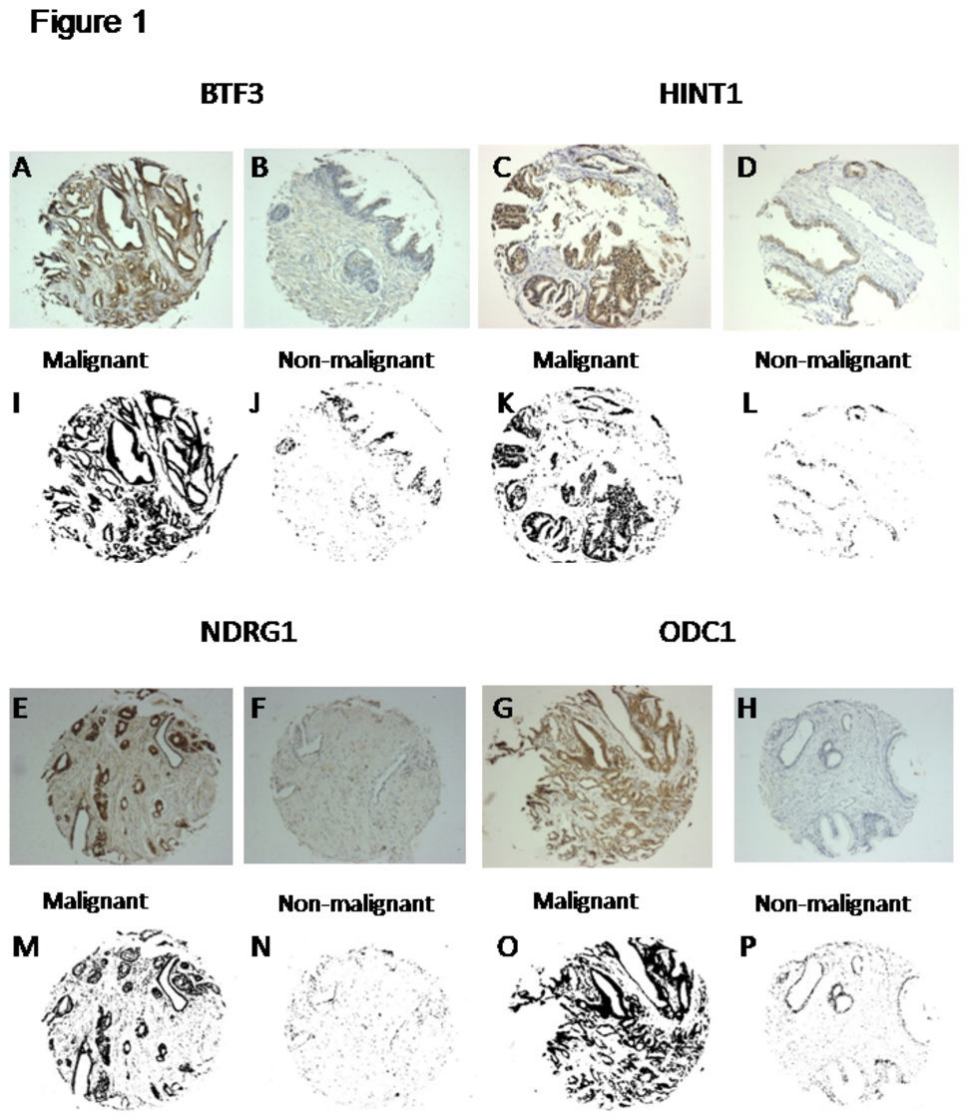
DAB-HRP tissue staining for BTF3, HINT1, NDRG1 and ODC1 in representative malignant (*A*,*C*,*E*,*G*) and non-malignant (*B*,*D*,*F*,*H*) prostate tissue cores (of over 500 tissue cores stained for each antibody) from human prostate arrays. These are representatives of over 450-500 tissue core images analyzed for each biomarker antibody using the automated image analysis protocol (see Materials and Methods – Image particle analysis). Each core was imaged using a Leica upright microscope (10x) and saved as a jpg file. Each image was used to calculate protein expression (DAB label, brown, largely in acinar cells). Colored RGB images (A-H) were converted to corresponding grayscale images (I-P) for the quantification of the DAB signal using Analyze Particle protocol in ImageJ software to obtain Total Area stained, in pixel. Protein expression for BTF3, HINT1, NDRG1 and ODC1 was increased in malignant v benign cores (p<0.0001).

**Figure 2:**
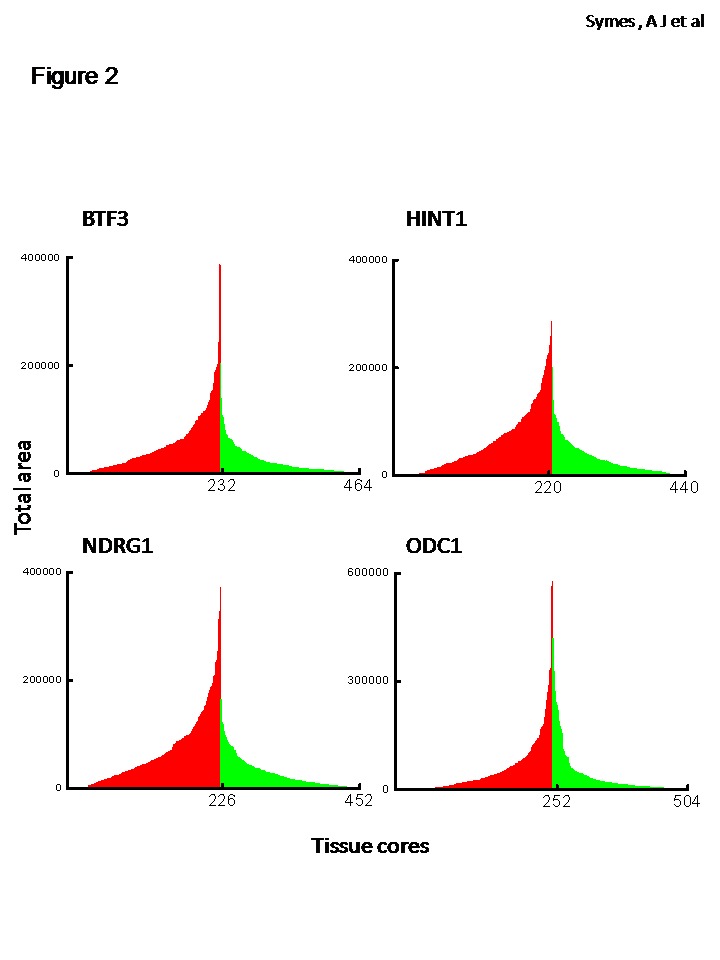
Quantitation of DAB signal in prostate tissue array. An unbiased, automated protocol was used to calculate Total Area stained (in pixel) standardized to amount of tissue in each core (see Methods). TA is converted to Area Fraction (total area divided by the total pixels in the image). Each bin is data for an individual, malignant (red) or non-malignant tissue (green) core. There is a significant increase in the AUC of protein expression in malignant v non-malignant prostate tissue (p<0.0001).

Expression of BTF3, HINT1, NDRG1 and ODC1 was largely epithelial and increased in malignant compared to benign or non-malignant prostate cores (Figure 1). We quantified the grayscale DAB-label (Figure 1 and Figure S1), in an unbiased manner, by using a reproducible, semi-automated particle analysis (Analyze Particles) protocol [8] using grayscale images of the stained tissue from over 450-500 individual prostate tissue cores for each biomarker (Figure 2). Calculations of total area and area fraction are given in Table 1. Integration of AUC revealed an increase in the expression of BTF3, HINT1, NDRG1 and ODC1 in malignant cores, diagnosed by histopathology, compared to non-malignant cores (p<0.0001, Mann Whitney U test). These results identify BTF3, HINT1, NDRG1 and ODC1 as proteins that are over-expressed in prostate cancer.

**Table 1:** Quantitation of protein expression in malignant and non-malignant human prostate tissue arryas using ImageJ software.

**Protein**	**Condition (*n*)**	**Total Area**	Area Fraction^*^	**Fold increase**
**BTF3**	Benign (236)	22301 ± 1697	1.66 ± 0.12	
	Malignant (228)	54750 ± 3588	4.09 ± 0.27	_2.5_
**HINT1**	Benign (214)	28564 ± 1882	2.13 ± 0.14	
	Malignant (225)	67173 ± 3977	4.93 ± 0.29	2.3
**NDRG1**	Benign (230)	27752 ± 1875	2.07 ± 0.14	
	Malignant (223)	72297 ± 4380	5.4 ± 0.33	2.6
**ODC1**	Benign (261)	38621 ± 4184	2.80 ± 0.31	
	Malignant (243)	65428 ± 5389	4.88 ± 0.4	1.8

DAB label, representing protein expression for BTF3, HINT1, NDRG1 and ODC1 was quantified in an unbiased manner, by using a reproducible, semi-automated particle analysis (Analyze Particles) protocol with ImageJ software. Over 500 individual prostate tissue cores (see methods) RGB images were converted into 16 bit grayscale. The results are means ± SE for the calculated parameters of total area and area fraction. These data are standardized for amount of tissue on each core by using the inverse protocol on ImageJ (see methods). Fold increase in the expression of protein in malignant compared to non-malignant (benign) cores was confirmed by AUC calculations of data from Figure 2. (n)= number of usable cores included in the analysis. ^*^Statistical analysis: expression, for all proteins, is significantly different in malignant compared to non-malignant prostate at P<0.0001 using Mann Whitney U test.

**Figure 3:**
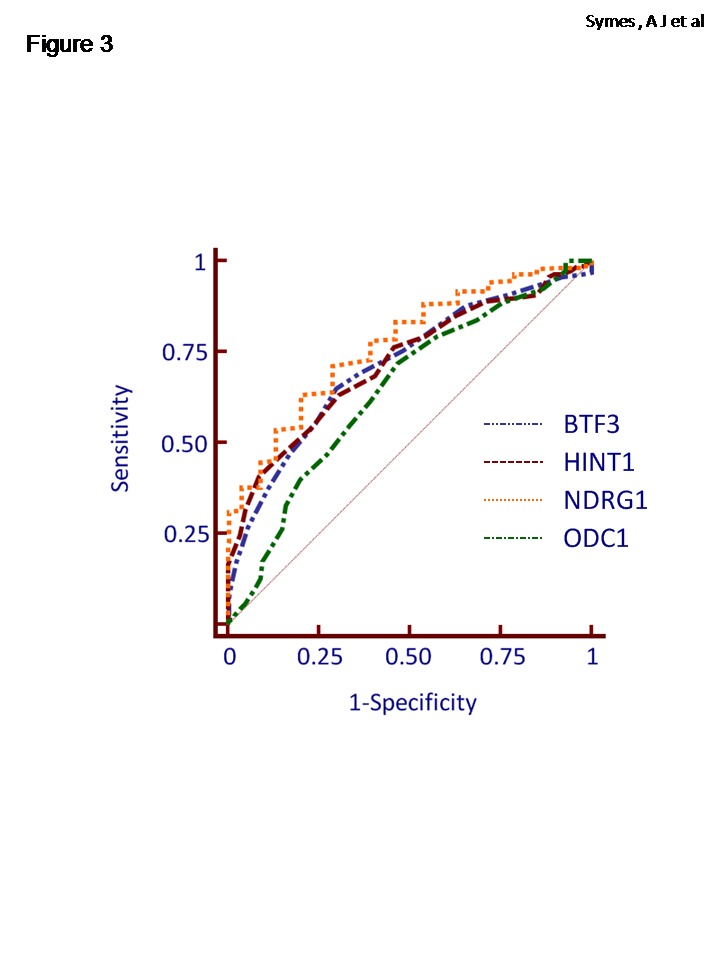
ROC of putative prostate cancer biomarkers. ROC curve demonstrates the discriminating performance of the protein expression in the differentiation between malignant and non-malignant tissue cores using the area fraction (probit) data for BTF3, HINT1, NDRG1 and ODC1. The operating characteristic values are given in Table S1. The solid line represents the ROC area of 0.5.

To investigate the utility of the proteins as putative biomarkers for prostate cancer diagnosis, we calculated the true positive rate (sensitivity) and false positive rate (1-specificity) by ROC curve (Figure 3). The area fraction data was transformed into probit and fitted to a Gaussian function. AUC values of 1.0 for an ROC curve represent high selectivity and sensitivity, whereas 0.5 suggests that the tested marker cannot distinguish between non-malignant and malignant categories. The dot plots of the transformed data are given in Figure S2. Sensitivity and 1-specificity were calculated for the area fraction values for NDRG1, BTF3, HINT1 and ODC1 (Table S1). Fixed threshold (criteria >) values for putative biomarkers for diagnosis range between 0.68 and 0.74 (Table S1), indicating high sensitivity for NDRG1, BTF3 and HINT1, as diagnostic markers for identifying prostate cancer in tissue cores by unbiased, quantitative immunohistochemistry. Positive likelihood ratios (LR+) for each biomarker at the designated criteria (Table S1) ranged from 1.7 to 2.4. A likelihood ratio of greater than 1 indicates that the result is associated with the presence of disease [15]. NDRG1, BTF3 and HINT1 also showed LR+ of >10 at various criteria cut off points; LR+s above 10, for a diagnostic test, are considered to provide strong evidence to rule in disease [15,16] . The diagnostic power was further enhanced by incorporating data from two biomarkers into a logistic regression model (Table 2). Gleason grades 4+3 and 3+4 made up >80% of the malignant tissue samples arrayed on the tissue array (between 221-241 tissue cores, see Table S2). There was no significant difference in expression of the four biomarkers when segregated for analysis based upon Gleason grade (4+3 and 3+4; Figure S3).

**Table 2:** Logistic regression model to identify combination of markers that may improve diagnostic power.

**Protein combination**	% cases correctly identfied*		
BTF 3 and HINT1	72		
BTF3 and NDRG1	78		
BTF3 and ODC1	93		
HINT1 and NDRG1	81		
HINT1 and ODC1	85		
NDRG1 and ODC1	97		

A logistic regression model was employed using MedCalc software to analyze the relationship between one dichotomous dependent variable (non-malignant v malignant) and one or more independent variables (two biomarkers). The outcome (percentage of cases correctly identified) suggests that within the cohort of samples used in this study, the diagnostic power to identify malignant cases is increased with the combination of BTF3 and ODC1 and NDRG1 and ODC1. *The significance level of the overall model fit for the analysis for all combinations is p<0.0001.

### Disease stratification using multiple markers and immunofluorescence staining

**Figure 4:**
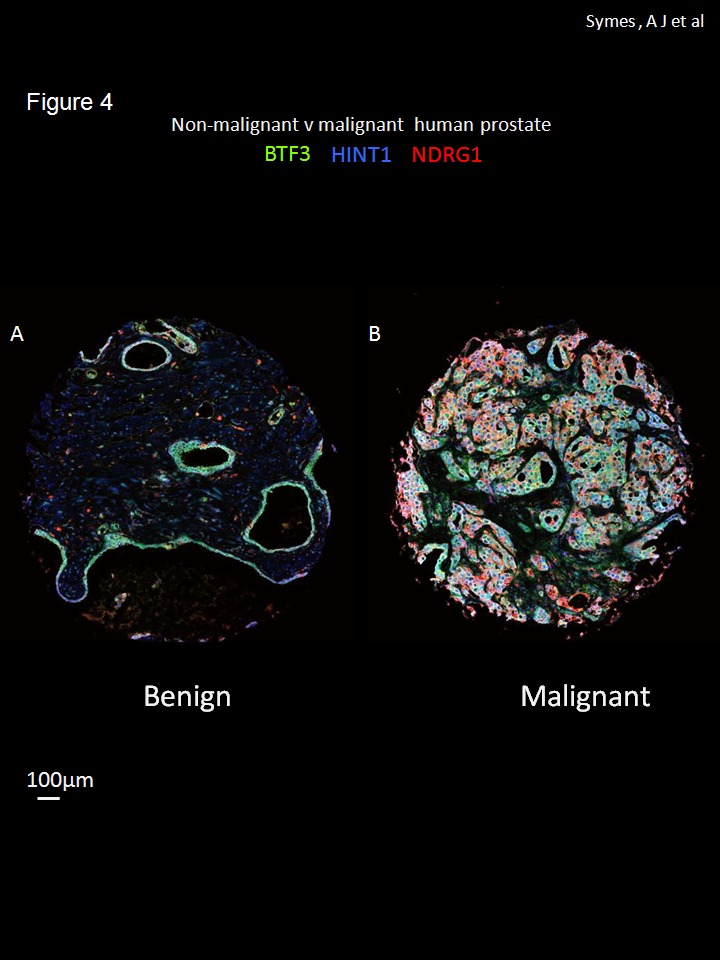
Multi-labeled immunofluorescence for BTF3, HINT1 and NDRG1. Co-expression of three proteins BTF3 (FITC-green), HINT1 (Cy3- blue) and NDRG1 (Cy5-red) in non-malignant (A) and malignant prostate tissue cores (B); images are representative tissue cores from the tissue array with over 200 samples. The three antibodies were incubated simultaneously and labeled with three Cy5 –red different fluorophores using Bond automated staining system. Whole tissue cores were imaged using a Leica confocal system and 4x4 tiling at exactly the same settings for comparison. Images were zoomed 3x using digital zoom in ImageJ. Scale bar =100µm.

It is estimated that around 30% of patients undergoing radical prostatectomy for clinically localized disease will experience biochemical relapse (defined as detectable PSA ≥ 0.2 ng ml^-1^ within 2 years of surgery). A cohort of patients used for the tissue array in this study, had undergone biochemical relapse [17]. We hypothesized that not only a change in expression but also co-localization of the identified biomarkers changes in prostate cancer epithelium. This was investigated by multi-labeled immunofluorescence staining using BTF3, HINT1 and NDRG1 antibodies (Figure 4). The 3 proteins were differentially expressed in non-malignant (Figure 4A) compared to malignant (Figure 4B) cores and found to be largely in the prostate epithelium, confirming the data from individual antibodies staining using 3,3-diaminobenzidine-horse radish peroxidase (DAB-HRP, Figure S1 and Figure 1). These results indicate not only that the expression of these protein biomarkers, individually, is increased in prostate cancer (Figure 2) but that the expression is largely epithelial and thus specific to the disease process (Figure 4). 

**Figure 5:**
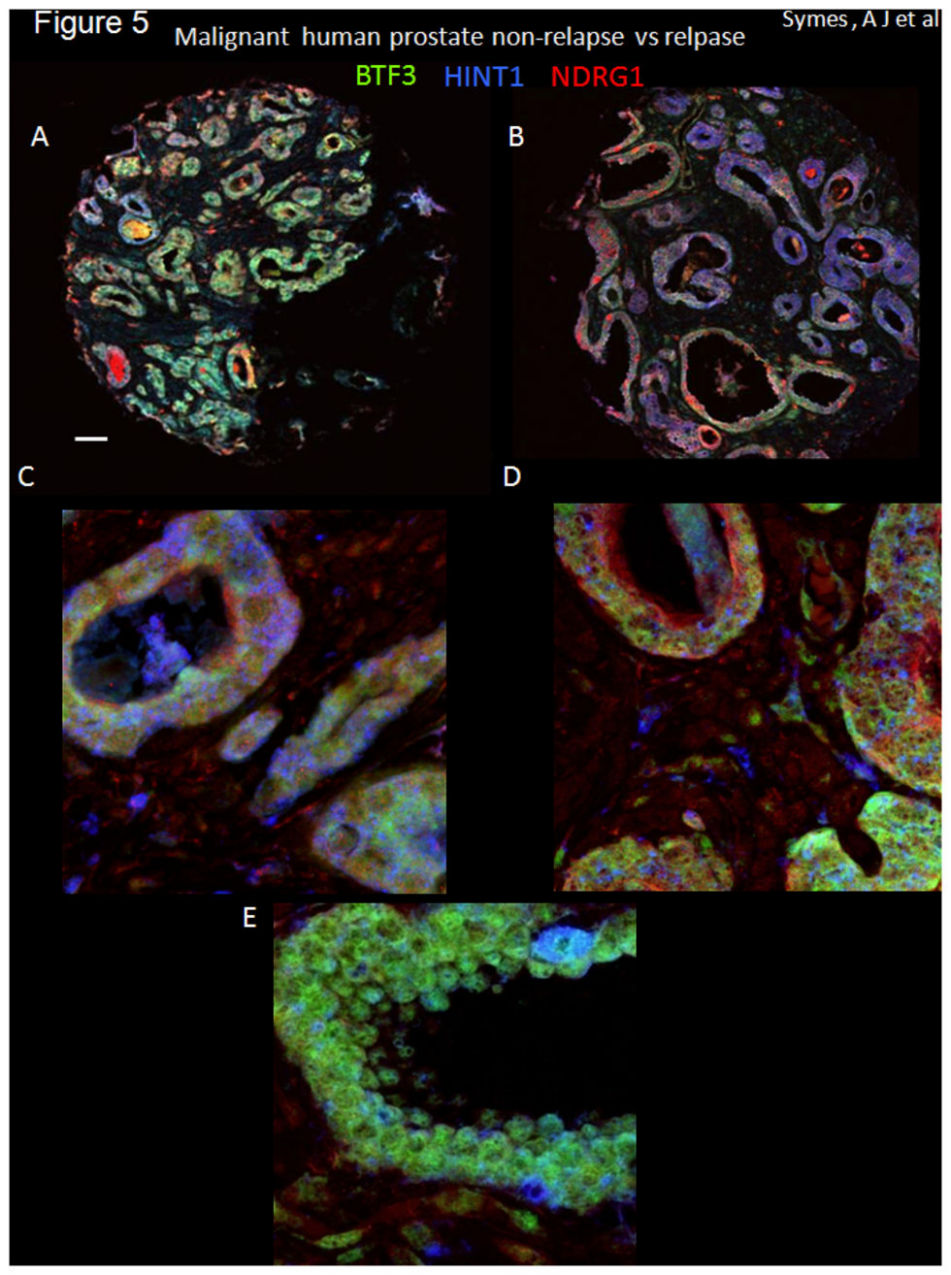
Differential pattern for BTF3, HINT1 and NDRG1 co-expression in malignant, biochemical relapse and non-relapse sample. Co-expression of three proteins BTF3 (FITC-green), HINT1 (Cy3- blue) and NDRG1 (Cy5 –red) in malignant prostate tissue cores (A to D). Composite micrographs of immunofluorescently stained prostate tissue cores from patients with biochemical relapse (defined as PSA ≥ 0.2 ng/ml within 2 years of radical prostatectomy). Tissue cores from 4 patients (matched pairs for Gleason score and PSA), non-relapsed (A) and biochemical relapse (B) representative images used for quantitative co-localization analysis (see methods) are shown. Imaging was performed using a Leica confocal microscope; A and B are low magnification; C and D higher magnification; E is an example of the expression of the three markers in non-malignant tissue also at higher magnification. The expression of BTF3 (green), for example is evident in non-relapsed samples whereas it not very evident in cores from relapsed patients. Imaging was performed using an Olympus confocal microscope (40x tiled (A and B) or 40x with digital zoom 3.5-4 (C, D and E). High magnification images (C,D and E) were deconvolved using Huygens Profession software. 16bit tif files for each fluorophore (FITC, Cy3 and Cy5) were imported into ImageJ and pseudo colored (FITC, green, Cy3, blue and Cy5, red). Z-projections for up to 27 images are shown for each tissue core. Scale bar =100µm for A and B and 20µm for C and D. Quantitative colocalization analysis of images (e.g. Figure 5C and D) showed that most significant change in the pattern of expression, in non-relapsed vs relapsed samples, was due to the change in co-expression of BTF3 (FITC, green) and HINT1 (Cy3, blue) with Pearson coefficients for colocalization for non-relapse vs relapse = 0.73 ± 0.02 vs 0.60 ± 0.07 (means ± SD, n=4 , significance of difference p<0.02).

The co-localization of BTF3, HINT1 and NDRG1 was next investigated in the multi-labeled immunofluorescence tissue cores from relapsed and non-relapsed patients from composite fluorescent images (Figure 5; BTF3, HINT1 and NDRG1, green, blue and red, respectively) in order determine the utility of this approach for prostate cancer stratification. There is an evident and quantifiable change in the pattern of staining of these protein biomarkers in biochemical relapse tissue cores: BTF3 expression appears less in relapsed, compared to non-relapsed samples, whereas HINT1 and NDRG1 expression appears dominant in relapsed tissue samples (representative tissue cores from biochemical relapse and control patients are shown in Figure 5A and B); quantitative colocalization analysis of high magnification deconvoluted images (Figure 5C and D) showed that most of the apparent change in the pattern of expression, for multi-labeled tissue sections in non-relapsed vs relapsed samples, was due to the change in co-expression of BTF3 (fluorescein isothiocyanate, FITC, green) and HINT1 (cyanin 3, Cy3, blue). Calculated Pearson coefficients for colocalization for non-relapse vs relapse were 0.73 ± 0.02 vs 0.60 ± 0.07 (means ± SD, n=4 , significance of difference p<0.02). Quantitative co-localization indicates that multi-labeling approach could be used for the stratification of prostate cancer in tissue cores.

## Discussion

By using global gene expression analysis, tremendous progress has been made in the identification of dysregulated genes in prostate cancer over the past decade. What has been lacking is the translation of these genes into clinically useful biomarkers for the diagnosis and stratification of the disease. Human prostate was one of the first tissues to be investigated for global changes in gene-expression in disease almost a decade ago. Incidentally, the approval of new markers by Food and Drug Administration, USA, particularly protein markers, has declined over the past decade. This decline could be attributed to three major problems in the identification and development of tissue based protein biomarkers for the diagnosis and prognosis of prostate cancer: 1. Lack of specificity, ie not epithelium specific (the site of initiation of the disease) 2. Lack of an unbiased, automated, quantitative analysis of protein expression that could identify robust, reprdoucible data for putative biomarkers 3. Lack of quantitative, proteomic approaches to for a molecular basis of prostate cancer prognosis.

Immunohistochemical markers could be extremely useful in primary or confirmatory diagnosis in support of histopathology, but due to the difficulties in identification or validation [18] only a handful of such markers exist. It also appears likely that histopathological analysis will remain a bulwark for prostate cancer diagnosis in the near to medium future. Thus in addition to continuing search for the identification of non-invasive biomarkers that could be detected in bodily fluids, it is important to keep refining and improving the immunochemical tissue biomarkers that could facilitate diagnosis, improve prognosis and provide useful predictive stratification of prostate cancer. Due to the increase in computing power over the last decade two technologies have matured that could help achieve these objectives. These are discussed below.

Morphological analysis is routinely used to diagnose prostate cancer, although IHC is used to assist morphological diagnosis, particularly in equivocal cases, e.g. in tissue samples from needle biopsies, transurethral resection specimens and metastatic tumor samples [19]. A complicating issue hindering discovery of new biomarkers for diagnosis and disease stratification of prostate (and other) cancer(s) from biopsy samples, has been a dearth of unbiased, quantitative methods to detect significant changes from IHC experiments for biomarker identification. There are, however, some exceptions to this approach. Rubin and colleagues [20] devised a quantitative approach to identify α-methylacyl-CoA racemase (AMACR). Initial studies showed almost uniform expression of this protein in cancerous tissue but in later analysis the expression was found to be less and, with reservation, this protein is used as a diagnostic marker.

To address the issue of bias and reliance on subjective assessment of expression of a given biomarker, we developed a method, free of experimenter bias, to quantify protein expression in prostate cancer [8] using a freely available software [21]. Unlike the conventional, scoring methods (e.g. of DAB staining) to estimate expression in tissue arrays, our approach allows unbiased quantitation of the total DAB signal. Although quantifying total DAB signal may not be ideal, by standardizing signal measurement and eliminating arbitrary scoring we identified BTF3, NDRG1, HINT1 and ODC1 protein levels to be over-expressed in prostate carcinoma, quantifiably and reproducibly, confirming the gene over-expression observed from our preliminary gene list. Operating characteristics (Figure 3 and Table S1) for BTF3, HINT1 and NDRG1 show AUC of 0.71 to 0.76; an AUC of >0.7 is considered acceptable for a diagnostic marker [22] indicating that, individually, these are adequate biomarkers of disease. The diagnostic power may be further enhanced when these markers are analyzed for aggregated diagnostic power (Table 2). Furthermore, BTF3, NDRG1 and HINT1, in quantitative co-localization studies (Figure 5) appear capable of discriminating between biochemical relapse and non-relapse prostate cancer. The biomarkers identified here are novel for their use in diagnosis and also in the combinations described for biochemical relapse of prostate cancer. The possible mechanisms by which these putative prostate carcinoma biomarkers may contribute to carcinogenesis are discussed below.

BTF3 is a 27kDa protein that forms a stable complex with RNA polymerase IIB and is required for transcriptional initiation [23] and known to be over-expressed in cells from pancreatic ductal carcinoma both *in vitro* and *in situ* [24]. NDRG1 (previously known as *Cap43*), a stress response gene, has been implicated in various cellular processes in health and disease. Unlike the expression of other biomarkers presented here, the expression of NDRG1 has been investigated in prostate tissue but is a subject of controversy with reports that it is increased [25] and decreased [26] in prostate cancer. It is important to note however that none of these studies employed an unbiased quantitative technique and therefore the expression analysis may be the reason for these discrepancies. 

We have previously shown that Wnt signaling pathway plays an important role prostate cancer [8,27] . NDRG1 has also been shown to influence Wnt signaling and there is a *prima facie* case for the involvement of NDRG1 in the regulation of Wnt signaling in prostate cancer cell lines [28]. NDRG1 gene is also an ETS-family fusion partner in ERG-expressing prostate cancer but unlike TMPRSS2-ERG and SCL45A3-ERG fusions, prevalent in prostate cancers, the NDRG1-ERG fusion is thought to encode for a chimeric protein [29]. No information exists regarding NDRG1 protein expression in prostate cancer; whether found to be fused to ERG or not, NDRG1 could be a useful protein biomarker for prostate cancer. 

HINT1 belongs to the histidine triad (HIT) family and a protein kinase C interacting protein [30]. No information exists on HINT1 expression or function in prostate cancer although it may act as tumor suppressor in mice [31]. In a hepatoma cell line, HINT1 inhibits activity of Wnt/ß-catenin signaling and gene transcription via TCF4 [32]. We have shown, previously, that there is a suppression of ß-catenin/TCF4 mediated gene transcription of TCF4 transcription targets [8]. It is tempting to speculate that HINT1 may contribute towards the inhibition of Wnt/ß-catenin signaling in prostate cancer [8]. 

L-Ornithine decarboxylase 1 (ODC1) is an enzyme in the polyamine synthesis pathway [33] a key factor in normal cell proliferation and neoplastic growth. ODC1 has been identified as a genetic marker for colon cancer [34] and over-expression of ODC1 has been linked to high risk neuroblastomas [35] and colorectal and breast cancers [36]. These data correlate to *in vivo* experiments with ODC1 knock-out mice which developed fewer tumors in response to various carcinogenic promoters in line with the reduced gene copy number [37]. 

Our data suggests that using an unbiased, quantitative approach to identify epithelium specific biomarkers in combination with immunofluorescence could prove useful in diagnosis, stratification and prognosis of prostate carcinoma. 

## Materials and Methods

### Prostate tissue array

Patient selection, disease state and construction details of tissue blocks are given elsewhere [8,17]. Briefly, tissue blocks were constructed using archival formalin-fixed, paraffin-embedded radical prostatectomy specimens from the 82 patients with pathological stage pT3a or b and pre-operative PSA stage of >3. 41 case pairs were matched for the following categories: pathological stage, Gleason grade and preoperative prostate-specific antigen (PSA) concentration. One patient in each pair had biochemical recurrence (defined as PSA ≥ 0.2 ng mL-1 within 2 years of surgery) and the other remained biochemically free of disease (defined as undetectable PSA at least 3 years after surgery). The cores were diagnosed (as benign or non-malignant and malignant) and marked by a pathologist and 5-6μm sections were cut from the tissue arrays onto coated slides. To eliminate bias, the experimenters were blind to staining, imaging and analysis, with the latter being automated (see below). Clinical details of the tissue samples used are given in Table S2 and [17]; pathologists scored a majority of samples (>80%) as Gleason grade 4+3 and 3+4. A sample size calculation was made for differentiating between over-expression using DAB-IHC; the sample size for the protein expression was calculated prior to the construction of the tissue array (with an α and ß of 0.05 and expected difference of 2.5 folds between the means of malignant and non-malignant samples to be 41 patients per group).

### Ethics approval

Ethical approval was given by the Joint UCL/UCLH committees on the ethics of human research. The review board (RB) approved the use of human tissue for prostate cancer research, in compliance with the International Committee on Harmonization of Good Clinical Practice (ICH GCP). For tissue array construction the samples were anonymized during the construction of the tissue arrays [17] and used for various immunohistochemical studies in a project approved by the UCL/UCLH ethics committee. The RB waived the need for informed consent because the samples used for tissue array were archival pathological samples.

### 3,3-diaminobenzidine-horse radish peroxidase(DAB-HRP) staining

 All staining was performed using Bond automated system [8] according to manufacturer’s protocols. Details of the staining procedure for IHC using DAB and antibodies (BTF3, HINT1, NDRG1 and ODC1) used are given in Methods S1. 

### Immunofluorescence staining using 3 antibodies in prostate tissue arrays

The protocol for immunofluorescence staining was similar to that described for DAB staining, above, except that BTF3, HINT1 and NDRG1 antibodies incubated simultaneously and stained with FITC, Cy5 and Cy3 respectively, according to manufacturer’s protocol. Images were acquired using a Leica 710 confocal microscope with an EC Plan-Neofluar (40x) objective. 

### Image particle analysis using ImageJ software

An unbiased, reproducible, automated particle analysis method [8] was employed to quantify the DAB signal on non-malignant (benign) and malignant human prostate tissue cores using ImageJ software [21]. Macros were written to execute the following sequence of events for acquired jpeg images: 1. Open image 2. Convert to 16-bit image 3. Set threshold 4. Analyze particle (Size 0.5- Infinity, Circularity 0.00-1.00) 5. Save image 6. Save particle information (count, total area, average size and area fraction) into an excel spreadsheet (rsb.info.nih.gov/ij/docs/pdfs/examples.pdf). Units are default ImageJ setting (pixels). Standardization for the amount of tissue per core was also quantified by using the inverse function (EditInvert image) in ImageJ with the protocol described above. Quantified DAB signal is expressed as signal / amount of tissue in each tissue core. A flow chart for DAB signal quantitation is given in Figure S1. For all antibodies tested, expression was observed to be largely epithelial and analysis was also restricted to the epithelial expression; set threshold parameters were chosen after manual analysis of random cores for the subsequent quantitation of the signal. A contiguous spreadsheet for all the usable cores (between 211 and 260 cores) for non-malignant vs malignant comparison, for different antibodies was constructed. Tests for normal distribution (Kolmogorov-Smirnov test) of the acquired data and subsequent statistical tests for significance of difference between protein expression in malignant and non-malignant cores was conducted using Mann Whitney U test. Mountain plots were constructed and analyzed for area under the curve (AUC) using Origin (Microcal). Receiver Operating Characteristics (ROC) curves were constructed after converting the data to normal distribution using probit using MedCalc software; likelihood ratios were also calculated from the ROC curves using MedCalc software. 

For quantitative colocalization of BTF3, HINT1 and NDRG1 tissue cores were imaged using an Olympus IX81 confocal system using a dry 40x objective (3.5 to 4x digital zoom). The ‘oif’ files were imported into Huygens Professional software for image deconvolution. The deconvolved images were saved as 16bit TIFF files for each channel (excitation/emission (nm) FITC = 488 / 519, Cy3 = 559 / 567, Cy5 = 635 / 664). The 16bit TIFF files were imported into ImageJ and the colocalization plugin (Wright Cell Imaging Facility, University of Toronto) used to calculate Pearson co-efficient for colocalization.

## Supporting Information

Figure S1 **Schematic representation of the process to calculate DAB signal and amount of tissue per sample (core) using ImageJ particle analysis protocol.** The DAB stained image (A) is converted to a grayscale image (B). For DAB signal calculation (C) a threshold is applied and the particles quantified. To calculate the amount of tissue, the grayscale image is inverted (D) and particles calculated.
(TIF)

Figure S2 **Dot plots of area fraction values for BTF3, HINT1, NDRG1 and ODC1 used to obtain operating characteristics (Figure 4).** N-M = non-malignant and M = malignant prostate tissue cores.
(TIF)

Figure S3 **Malignant prostate tissue cores (221-241) were analyzed on for protein expression (area fraction (AF) / amount of tissue) based upon the Gleason grade.** Majority (>80%) of the malignant tissue cores were graded 4+3 and 3+4. A comparison between these two Gleason grades did not show a significant difference for the expression of four biomarkers tested (Mann-Whitney test). Data is presented as means ± SEM for 75-109 individual tissue cores for each grade for the four biomarkers.
(TIF)

Methods S1 (DOC)

Table S1 (PDF)

Table S2 (PDF)
