# Correction: Quantitative Analysis of BTF3, HINT1, NDRG1 and ODC1 Protein Over-Expression in Human Prostate Cancer Tissue

**DOI:** 10.1371/annotation/80c6bb6d-657b-46be-ba77-3de5d528c89e

**Published:** 2014-01-10

**Authors:** Andrew J. Symes, Marte Eilertsen, Michael Millar, Joseph Nariculam, Alex Freeman, Maria Notara, Mark R. Feneley, Hitendra R. H. Patel, John R. W. Masters, Aamir Ahmed

The name of the eighth author is spelled incorrectly. The correct name is: Hitendra R. H. Patel.

