# Supplementary figures and images for: Quantitative Analysis of BTF3, HINT1, NDRG1 and ODC1 Protein Over-Expression in Human Prostate Cancer Tissue
Source: PLoS One. 2013 Dec 27;8(12):e84295. doi: 10.1371/journal.pone.0084295 (PMC3874000; doi:10.1371/journal.pone.0084295)

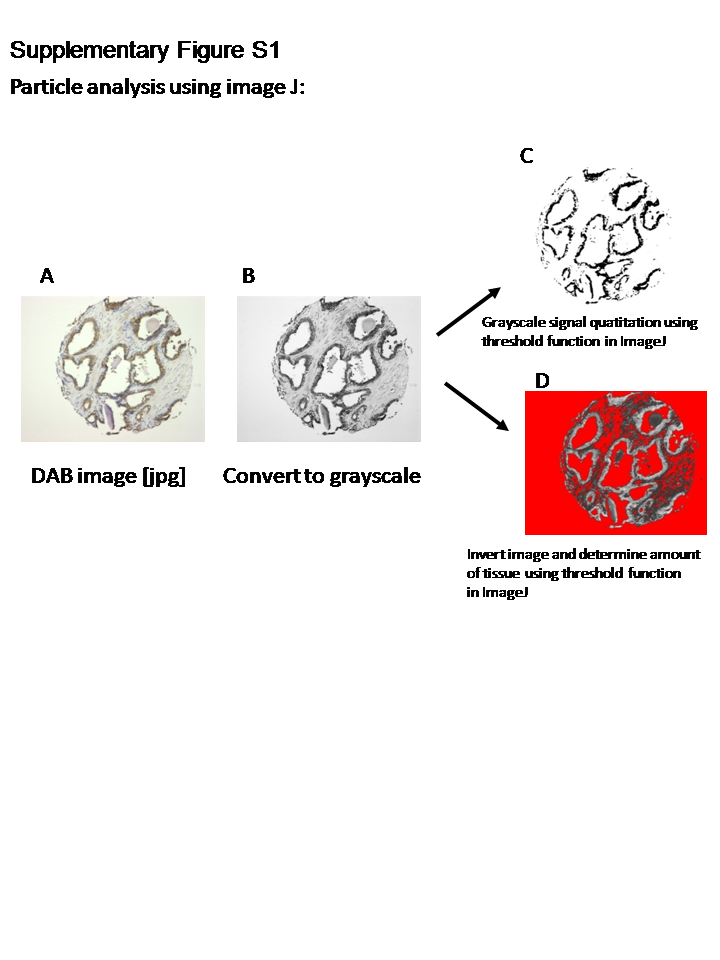

Supplement: Figure S1 — Schematic representation of the process to calculate DAB signal and amount of tissue per sample (core) using ImageJ particle analysis protocol. The DAB stained image (A) is converted to a grayscale image (B). For DAB signal calculation (C) a threshold is applied and the particles quantified. To calculate the amount of tissue, the grayscale image is inverted (D) and particles calculated. (TIF) [file pone.0084295.s001.tif]

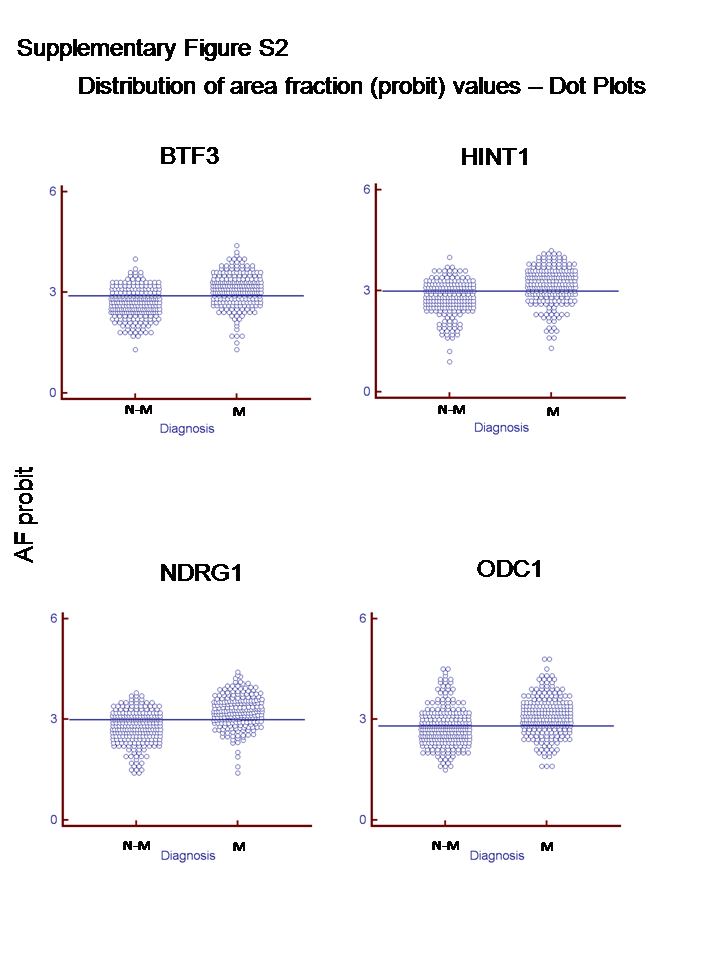

Supplement: Figure S2 — Dot plots of area fraction values for BTF3, HINT1, NDRG1 and ODC1 used to obtain operating characteristics (Figure 4). N-M = non-malignant and M = malignant prostate tissue cores. (TIF) [file pone.0084295.s002.tif]

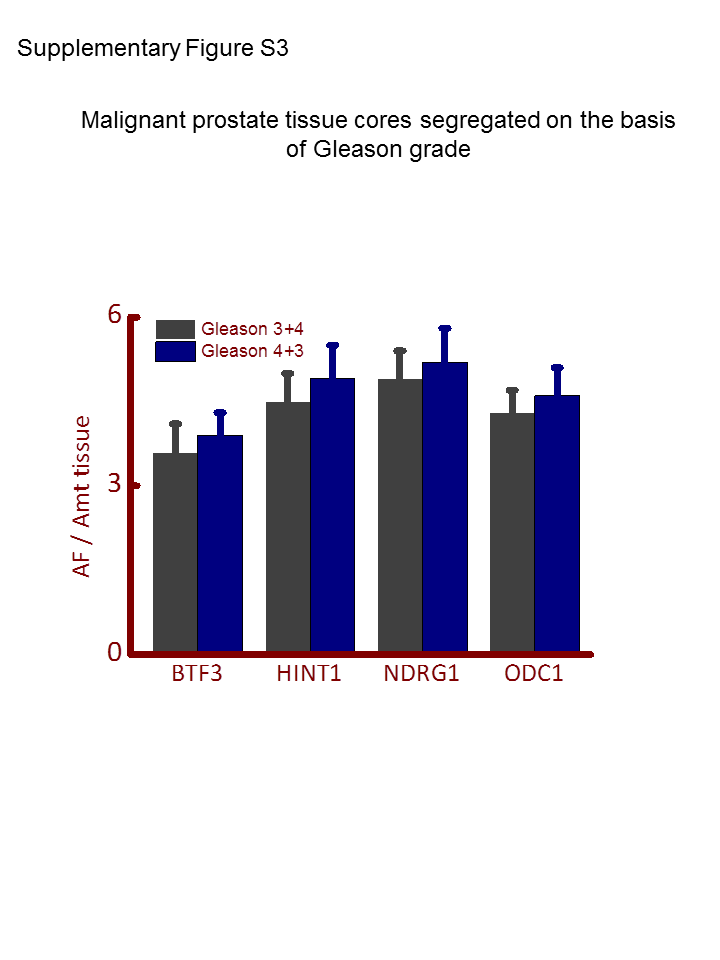

Supplement: Figure S3 — Malignant prostate tissue cores (221-241) were analyzed on for protein expression (area fraction (AF) / amount of tissue) based upon the Gleason grade. Majority (>80%) of the malignant tissue cores were graded 4+3 and 3+4. A comparison between these two Gleason grades did not show a significant difference for the expression of four biomarkers tested (Mann-Whitney test). Data is presented as means ± SEM for 75-109 individual tissue cores for each grade for the four biomarkers. (TIF) [file pone.0084295.s003.tif]
