# Supplementary material for: Quantitative Analysis of BTF3, HINT1, NDRG1 and ODC1 Protein Over-Expression in Human Prostate Cancer Tissue
Source: PLoS One. 2013 Dec 27;8(12):e84295. doi: 10.1371/journal.pone.0084295 (PMC3874000; doi:10.1371/journal.pone.0084295)
Supplement: Methods S1 — (DOC) [file pone.0084295.s004.doc]

**DAB-HRP staining**

All staining was performed using Bond automated system according to manufacturer’s protocols. Briefly, tissue arrays were dried overnight at 60°C, prior to performing antigen retrieval using pressure cooker (5 min full pressure and 20 min in Novocastra pH 6 retrieval buffer. All immunostaining protocols were carried out on Vison Biosystems Bond X robot using Define (HRP-polymer) detection kits. H_2_O_2_ block, DAB (3,3-diaminobenzidine) and haematoxylin counterstain were performed as per manufacturer protocols. Images (1300 x 1030 pixels) for each tissue core were acquired at 10x magnification at standardized settings using a Leica microscope. All the antibodies were obtained from Abcam and used at the following concentrations [µg/ml] BTF3 [10], HINT1 [0.5], NDRG1 [25] and ODC1 [1].
