# Supplementary material for: Quantitative Analysis of BTF3, HINT1, NDRG1 and ODC1 Protein Over-Expression in Human Prostate Cancer Tissue
Source: PLoS One. 2013 Dec 27;8(12):e84295. doi: 10.1371/journal.pone.0084295 (PMC3874000; doi:10.1371/journal.pone.0084295)
Supplement: Table S1 — (PDF) [file pone.0084295.s005.pdf]

Supplementary Table S1: **True positive and true negative rates for putative prostate cancer diagnostic markers from ROC curve analysis**

**Biomarker**	**AUC**	**Criteria >**	**Sensitivity**	**1-Specificity**	**+ Likelihood ratios**
BTF3	0.74	2.7	0.74	0.6	1.8
HINT1	0.71	2.9	0.67	0.61	1.7
NDRG1	0.76	3	0.7	0.71	2.4
ODC1	0.67	2.8	0.68	0.62	1.8

Operating characteristics Sensitivity and 1-Specificity of the area fraction values for BTF3, HINT1, NDRG1 and ODC1 at a specific value (Criteria >) calculated using ROC curves. AUC = area under the curve. An AUC of >0.7 is considered to provide adequate discrimination for a diagnostic marker. 95% CI intervals (sensitivity; 1-specificity) are: BTF3 (0.69-0.8;0.5-0.63), HINT1 (0.61-0.74;0.52-0.65), NDRG1 (0.64-0.76;0.65-0.76), ODC1 (0.61-0.73;0.56-0.68). The data is tabulated from Figure 3.
