# Supplementary material for: Quantitative Analysis of BTF3, HINT1, NDRG1 and ODC1 Protein Over-Expression in Human Prostate Cancer Tissue
Source: PLoS One. 2013 Dec 27;8(12):e84295. doi: 10.1371/journal.pone.0084295 (PMC3874000; doi:10.1371/journal.pone.0084295)
Supplement: Table S2 — (PDF) [file pone.0084295.s006.pdf]

Supplementary Table S2. **Forty-one pairs of prostate cancer patients who had undergone radical prostatectomy, matched for pathological stage, Gleason sum score, preoperative PSA, and paired for relapse and non-relapse status. 82 patient samples were used in 41 pairs (adapted from reference (22), Nariculam, J et al, Asian J Androl 11: 109-118, 2009).**

Pair No.	Non-relapser Pathological	Relapser Gleason	Pre-operative PSA stage	Follow-up grade	Pathological stage	Gleason grade (years)	Pre-operative PSA
1	pT3a	3 + 4 = 7	9.7	5	pT3a	3 + 4 = 7	9.9
2	pT3a	3 + 4 = 7	6	5	pT3a	3 + 4 = 7	9.8
3	pT3a	3 + 3 = 6	7	5	pT3a	2 + 4 = 6	9
4	pT3a	2 + 3 = 5	18	5	pT3a	2 + 3 = 5	15
5	pT3a	3 + 2 = 5	16	5	pT3a	3 + 2 = 5	19
6	pT3a	4 + 2 = 6	13	5	pT3a	2 + 4 = 6	10.2
7	pT3a	3 + 3 = 6	9	5	pT3a	3 + 3 = 6	4.3
8	pT3a	4 + 3 = 7	9	5	pT3a	4 + 3 = 7	8
9	pT3a	3 + 4 = 7	8	5	pT3a	4 + 3 = 7	8
10	pT3a	4 + 4 = 8	16	5	pT3a	4 + 4 = 8	16
11	pT3a	3 + 4 = 7	17	5	pT3a	4 + 3 = 7	14
12	pT3a	4 + 3 = 7	10	5	pT3a	4 + 3 = 7	12
13	pT3a	3 + 4 = 7	5	5	pT3a	4 + 3 = 7	8
14	pT3a	4 + 3 = 7	6	5	pT3a	3 + 4 = 7	7
15	pT3a	3 + 4 = 7	8	5	pT3a	3 + 4 = 7	8
16	pT3a	3 + 4 = 7	8	5	pT3a	3 + 4 = 7	8
17	pT3a	3 + 2 = 5	7	5	pT3a	3 + 2 = 5	5
18	pT3b	4 + 3 = 7	13	5	pT3b	4 + 3 = 7	12
19	pT3a	4 + 3 = 7	18	3	pT3a	4 + 3 = 7	17
20	pT3a	3 + 4 = 7	6	3	pT3a	4 + 3 = 7	8
21	pT3a	3 + 4 = 7	4	7	pT3a	4 + 3 = 7	8
22	pT3a	3 + 4 = 7	5	7	pT3a	4 + 3 = 7	7
23	pT3a	3 + 4 = 7	6	3	pT3a	3 + 4 = 7	6
24	pT3a	3 + 4 = 7	10	3	pT3a	3 + 4 = 7	10
25	pT3a	3 + 4 = 7	17	7	pT3a	4 + 3 = 7	16
26	pT3a	3 + 4 = 7	6	3	pT3a	4 + 3 = 7	5
27	pT3a	3 + 4 = 7	14	3	pT3a	3 + 4 = 7	14
28	pT3a	3 + 2 = 5	6	7	pT3a	2 + 3 = 5	9
29	pT3a	3 + 4 = 7	7	3	pT3a	4 + 3 = 7	6
30	pT3a	3 + 4 = 7	11	3	pT3a	4 + 3 = 7	18
31	pT3a	3 + 4 = 7	13	3	pT3a	4 + 3 = 7	16
32	pT3a	3 + 4 = 7	6	3	pT3a	4 + 3 = 7	9

33	pT3a	3 + 4 = 7	9	8	pT3a	4 + 3 = 7	8
34	pT3a	3 + 4 = 7	14	3	pT3a	4 + 3 = 7	16
35	pT3a	3 + 4 = 7	20	3	pT3a	4 + 3 = 7	21
36	pT3a	4 + 4 = 8	15	10	pT3a	5 + 3 = 8	14
37	pT3a	3 + 4 = 7	7	10	pT3a	3 + 4 = 7	11
38	pT3a	3 + 4 = 7	10	7	pT3a	3 + 4 = 7	10
39	pT3b	4 + 3 = 7	13	3	pT3b	4 + 3 = 7	18
40	pT3b	3 + 4 = 7	8	3	pT3b	3 + 4 = 7	7
41	pT3b	4 + 3 = 7	22	10	pT3b	4 + 3 = 7	13
